# Development of rhesus macaque astrocyte cell lines supporting infection with a panel of viruses

**DOI:** 10.1371/journal.pone.0303059

**Published:** 2024-05-14

**Authors:** Stefanie Reiter, Ting Sun, Sabine Gärtner, Stefan Pöhlmann, Michael Winkler

**Affiliations:** 1 German Primate Center—Leibniz Institute for Primate Research, Infection Biology Unit, Göttingen, Germany; 2 Department of Neurogenetics, Max Planck Institute for Multidisciplinary Sciences (City Campus), Göttingen, Germany; 3 Faculty of Biology and Psychology, Georg-August-University Göttingen, Göttingen, Germany; CEA, FRANCE

## Abstract

Non-human primate (NHP)-based model systems are highly relevant for biomedical research. However, only few NHP cell lines are available and the generation of additional cell lines is an urgent need to help in the refinement and replacement of these models. Using lentiviral transduction of c-Fos, we established cell lines from the brain of rhesus macaques (Macaca mulatta). Transcriptome analysis revealed that these cell lines are closely related to astrocytes, which was confirmed by immunoblot and immunofluorescence microscopy detecting expression of the astrocyte marker glial fibrillary acidic protein (GFAP). Quantitative real-time PCR (qRT-PCR) demonstrated that major pathways of the interferon (IFN) system are intact. Using retroviral pseudotypes we found that the cell lines are susceptible to entry driven by the glycoproteins of vesicular stomatitis virus (VSV), lymphocytic choriomeningitis virus (LCMV) and to a lesser extent influenza A virus (IAV). Finally, these cells supported growth of Zika virus (ZIKV) and Papiine alphaherpesvirus 2 (PaHV2). In summary, we developed IFN-responsive cell lines from the rhesus macaque brain that allowed entry driven by several viral glycoproteins and were permissive to infection with ZIKV and a primate simplexvirus. These cell lines will be useful for efforts to analyze neurotropic viral infections in rhesus macaque models.

## Introduction

Macaques are important animal models for biomedical and infectious disease research, due to their physiologic similarities and their close phylogenetic relationship to humans. Consequently, macaque models of infectious diseases, such as infection with Ebola virus (EBOV) or Zika virus (ZIKV), closely mirror the pathogenesis observed in human patients [1–3]. However, the close relatedness to humans has also led to ethical concerns and a call for strategies to replace, reduce and refine (3R) [4] non-human primate (NHP) model systems. The development of suitable ex vivo models, including cell lines, will aid refinement and replacement.

The availability of NHP induced pluripotent stem cells (iPSC) [5–7] allows for generation of cells of different lineages. However, this approach is labor intensive and requires specialized and expensive reagents. In contrast, continuously growing cell lines offer the advantage of low cost and high scalability [8]. Since cell lines established from tissue explants usually have limited reproductive capacity, viral or cellular genes like the simian virus 40 (SV40) large T [9] or the catalytic subunit of telomerase reverse transcriptase (TERT) [10,11] have been employed to generate immortalized cell lines.

Although thousands of cell lines of human origin are available from commercial suppliers (such as ATCC), only few cell lines of NHP origin can be obtained. Furthermore, only few cell lines have been reported in literature [12–14] that are not yet available via repositories. Thus, there is a clear need for new cell lines of macaque origin. In particular, to our knowledge, no cell lines are available that have been derived from macaque neuronal tissue. Here, we report the generation of cell lines from macaque neuronal tissues, such as trigeminal ganglia, cortex and hippocampus, that are of astrocyte origin, as demonstrated by RNAseq analyses and expression of the astrocyte marker glial fibrillary acidic protein (GFAP). The interferon (IFN)-stimulated gene (ISG) MX1 was induced upon treatment with IFN or viral infection, demonstrating functionality of key pathways of the IFN system. The cells allowed for entry driven by glycoproteins from diverse viruses and supported productive infection with primate simplexvirus Papiine alphaherpesvirus 2 (PaHV2) and ZIKV.

## Materials and methods

### Ethics statement

The German Primate Center (DPZ) has permission for housing and breeding non-human primates under license number 392001/7 issued by the local veterinary authorities. Rhesus macaques were housed under conditions in accordance with the German Animal Welfare Act and the European Union guidelines on the protection of animals used for scientific purposes. For the purpose of this study, only leftover materials were used, which were derived from animals in experiments approved by an external ethics committee authorized by the Lower Saxony State Office for Consumer Protection and Food Safety (Niedersächsisches Landesamt für Verbraucherschutz und Lebensmittelsicherheit) (project license: 33.19-42502-04-18/2902).

### Plasmids and oligonucleotides

Plasmid HIV-gag-pol was a kind gift from Thomas von Hahn (Hannover). Plasmids for Murine leukemia virus (MLV) pseudotype production (MLV-luc, MLV-gag-pol) and expression of glycoproteins from Vesicular stomatitis virus (VSV-G), influenza A virus strain WSN (IAV HA and NA) and Lymphocytic choriomeningitis virus strain Armstrong (LCMV-GPC) have been described previously [15–17]. All oligonucleotides (Table 1) were purchased from Sigma-Aldrich (Steinheim, Germany).

**Table 1:** Oligonucleotides used for cloning.

Name	Sequence 5’-3’
LentdNr-for	AACCGAATTTTTTCCCACCCGGGTCTAATTCTCCCCCGCT
LentdNr-rev	AGCGGGGGAGAATTAGACCCGGGTGGGAAAAAATTCGGTT
LentdNo-for	ACCACCGCACAGCAAGCCGGCGCTGATCTTCAGACCT
LentdNo-rev	AGGTCTGAAGATCAGCGCCGGCTTGCTGTGCGGTGGT
neo3aarI	CGATCACCTGCAAGGGTACGATATCTCCGGATCAGAAGAACTC
EMCV_IRES3	GCTGAAGGATGCCCAGAAGG
ID2-5N	CCGCGGCCGCACCATGAAAGCCTTCAGTCCCGT
ID2-3X	CCCTCGAGTCAGCCACACAGTGCTTTGC
cFos-5N (6250	CCGCGGCCGCACCATGATGTTCTCGGGCTTCAA
cFos-3X (6179)	CACTCGAGTCACAGGGCCAGCAGCGTGG
16E7-5N	CCGCGGCCGCACCATGCATGGAGATACACCTAC
16E7-3X	GGCTCGAGTTATGGTTTCTGAGAACAGA
hcMyc-5B	GCGGATCCCTGGATTTTTTTCGGGTAGTG
hcMyc-3N	GGTGCGGCCGCTTACGCACAAGAGTTCCGTAG
BMI1-5N	CCGCGGCCGCACCATGCATCGAACAACGAGAAT
BMI1-3X	GCCTCGAGTCAACCAGAAGAAGTTGCTGA
cMyb-5N	CCGCGGCCGCACCATGGCCCGAAGACCCCGGCA
cMyb-3X	GTCTCGAGTCACATGACCAGCGTCCGGG
hKlf4-5B	GGGGATCCATGGCTGTCAGCGACGCGC
hKlf4-3N	GTGGCGGCCGCTTAAAAATGCCTCTTCATGTG
CCND1-5N	CCGCGGCCGCACCATGGAACACCAGCTCCTGTG
CCND1-3X	CCCTCGAGTCAGATGTCCACGTCCCGCAC
RhGABBR2-5X	CTCGAGCACCATGGCTTCCCCGCGGAGCTCC
RhGABBR2-3E	GAATTCTCAGGCCCGAGACCATGACTCG
RhGAD65-5X	CTCGAGCCACCATGGCATCTCCGGGCTCTGGC
RhGAD65-3E	GAATTCTTATAAATCTTGTCCAAGRCG
RhOLIG1-5X	CTCGAGCCACCATGTACTATGCGGTTTCCCAG
RhOLIG1-3E	GAATTCTCACTTGGAGAAYTGCGCCTG

For lentiviral transduction, we modified a pReceiver-Lv205-based expression plasmid (Genecopoeia, Rockville, MD, USA) to harbor the SV40 large T gene (lT) in a pQCXIP-based expression cassette. In a first step, pLenti-IP-PB1 was generated by insertion of a NdeI/Pfl23II fragment containing CMV promoter-PB1-IRES into EX-A2639-Lv205, as previously described [18]. In a second step, we inserted the SV40 large T gene as a NotI/PmlI fragment into pLenti-IP-PB1. The resulting plasmid pLenti-IP-large T contained the SV40 large T gene under control of the human cytomegalovirus (HCMV) enhancer/promoter followed by an internal ribosomal entry sequence (IRES) and a puromycin resistance gene.

To generate additional lentiviral vectors harboring immortalization genes, we modified the lentiviral plasmid pLenti6/V5-GW/LacZ stepwise into a vector with an expression cassette similar to pQCXIP and a multiple cloning site containing sites for NotI-BamHI-AgeI-HpaI-MluI-XhoI-MluI-EcoRI. First, a NdeI/Acc65I fragment harboring HCMV enhancer/promoter, multiple cloning site and an IRES with mutated AarI site was inserted into pLenti6/V5-GW/LacZ. In the next step, NruI and NotI sites in the lentiviral backbone were mutated by splice overlap PCR, using primers LentdNr-for/LentdNr-rev and LentdNo-for/LentdNo-rev (Table 1). The resulting vector was then cut with Acc65I and an Acc65I/AarI-fragment containing the neomycin resistance gene was inserted after amplification from pQCXIN-mcs [19] using primer neo3aarI/EMCV_IRES3 to obtain pLCXIN-mcs. Finally, the neomycin resistance gene was exchanged with a hygromycin resistance gene as an EcoRI/EcoRV fragment from pQCXIHy-mcs [19] to obtain pLCXIHy-mcs.

To obtain lentiviral vectors harboring the immortalization genes ID2, c-Fos (FOS), HPV16 E7, BMI1, c-Myb (MYB) isoform 3, and Cyclin-1 (CCND1) the genes were amplified from human cDNA or plasmid (pLXSN-16E6E7) using primers listed in Table 1 and cloned using NotI/XhoI sites into pLCXIHy-mcs. The genes c-Myc (MYC) isoform 1 and KLF4 isoform 2 had previously been cloned by PCR into pENTR3C (Invitrogen, Carlsbad, CA, USA) using primers listed in Table 1 and subcloned into pLCXIHy-mcs using BamHI+XhoI sites. All sequences were verified by sequencing to correspond to reference sequences stored in GenBank.

For expression of rhesus macaque GABBR2, GAD2 and Olig1 the genes were amplified from cDNA generated from rhesus macaque brain tissue using primers RhGABBR2-5X and RhGABBR2-3E, RhGAD65-3E and RhGAD65-5X or RhOLIG1-3E and RhOLIG1-5X, respectively (Table 1). PCR fragments were cloned into vectors pmScarlet-idNS-C1 (GAD2, Olig1) or pmScarlet-idNS-N1 (GABBR2) using XhoI and EcoRI sites. Vector pmScarlet-idNS-C1 was generated by mutating SalI and NotI sites within the mScarlet-i gene in original vector pmScarlet-i-C1 (kind gift from Dorus Gadella; Addgene plasmid #85044, Watertown, MA, USA). Vector pmScarlet-idNS-N1 was generated by replacing the EYFP gene in pEYFP-N1 (Clontech, Mountain View, CA, USA) with the modified mScarlet-idNS gene.

### Cell culture

The cell lines 293T (DSMZ GmbH, Braunschweig, Germany), Vero76 (kind gift by Andrea Maisner) and A549 (kind gift by Thomas Schulz) were cultivated in DMEM supplemented with 10% fetal calf serum (FCS) and penicillin/streptomycin. Cell lines were routinely tested for mycoplasma and the identity of human cell lines was verified by short tandem repeat (STR) analysis [20]. The hybridoma cell line D1-4G2-4-15 [21] was purchased from LGC/ATCC (Teddington, UK) and cultivated in RPMI1640 supplemented with 10% FCS and penicillin/streptomycin.

### Establishment of primary cultures

Tissue samples for trigeminal ganglia, brain cortex and hippocampus were obtained from two male animals sacrificed at age of 6 (animal 2817) and 7 (animal 2880) years for reasons unrelated to this project. Tissue samples were chopped into small (1 mm^3^) pieces, transferred to separate Eppendorf tubes und incubated in 1 mL PBS containing 5 mg Collagenase IV. Samples were incubated for 1–3 hours at 37°C and 800 rpm in a thermomixer. After centrifugation for 5 min at 300 x g, cells were resuspended in DMEM/F12 medium and seeded separately into 6-well plates in a total volume of 3 mL/well. All media were supplemented with 10% FCS and penicillin, streptomycin, gentamycin, nystatin and amphotericin B. After outgrowth during the next two weeks, cells were seeded for transduction with immortalizing genes.

### Virus

The primate herpesvirus PaHV2 (HVP2) strain X313 was a kind gift by David Brown and Matthew Jones, Public Health England. ZIKV strain MR766 was cloned from RNA and rescued by transfection of Vero76 cells [22]. Virus stocks were prepared on Vero76 cells after infection at low multiplicity of infection (MOI). Virus containing supernatant was harvested when complete cytopathic effect had developed, filtered through 0.45 μm filters to remove cell debris and stored at -80°C.

For infection with Vesicular stomatitis virus (VSV), we used a recombinant virus, VSV ncp*, which carries the eGFP gene as reporter (the asterisk stands for eGFP) and has four amino acid changes within the matrix gene rendering it less cytopathogenic (ncp) [23].

### Retroviral transduction

For production of transducing lentivirus, we seeded 293T cells in T25 flasks at about 10^6^ cells/flask. Cells were transfected the next day with 6 μg lentiviral vector, 3 μg HIV gag-pol(SCA) and 3 μg pHIT/G expressing VSV-G, using the calcium phosphate method, as previously described for retroviral vectors [24,25]. Plasmid HIV gag-pol(SCA), where amino acids 1–204 of the HIV capsid gene (hCA) were substituted by amino acids 1–202 of SIV capsid gene (sCA) was used for improved transduction of rhesus macaque cells [18]. After two days, cell culture supernatants were harvested, filtered through 0.45 μm filters and stored as aliquots at -80°C. Lentiviral stocks were not titrated directly, but in parallel we used a EGFP expressing lentivirus to experimentally determine that we were able to achieve titers between 10^6^−10^7^/mL on rhesus macaque kidney cells [18].

Transduction and selection was performed as recently described [26]. Briefly, cells were seeded at 5,000 cells per well in a 96-well plate. After overnight incubation, 50 μl of transducing lentiviral particles were added. For transduction of several immortalization genes, four separate particle preparations were pooled (pools arbitrarily labelled with letters, e.g. “g” or “r”), resulting in a total volume of 200 μl per well. On the next day, cells were transduced with a separate pool of lentiviral transducing particles, resulting in cells transduced with two different pools (labelled “gr”). Spinoculation at 4,000 × g for 30 min was used to increase the transduction efficiency. After two days, cells were detached and transferred into a 24-well plate containing selection medium. We used 50 μg/mL hygromycin and 2.5 μg/mL puromycin for selection of hygromycin (gr-pools) or puromycin (large T, lT) resistance, respectively.

During establishment of cell lines transduced with pools only a subset of transduced genes is actually present in the final cell line [27]. Therefore, successful transduction of cell lines with individual transgenes (gr-pools) was analyzed by PCR from genomic DNA isolated from these cell lines. For the PCR we used one common forward primer within the human cytomegalovirus promoter driving transgene expression and a gene specific reverse primer (Table 2).

**Table 2:** Oligonucleotides used for cloning.

Name	Sequence 5’-3’
BMI1-rev	CAGCAGAAGGATGAGCTGCA
cycD1-rev	AGGAAGCGGTCCAGGTAGTT
E7-rev	GATGGGGCACACAATTCCTA
FOS-rev	ACTGGTCGAGATGGCAGTGA
ID2-rev	TGGTGATGCAGGCTGACAAT
Klf4-rev	GCTCTCCAGGTCTGTGGCCA
Myb-rev	GGCACTGCACATCTGTTCGA
Myc-rev	GGCAGCAGCTCGAATTTCTT

### Immunoblot

For immunoblot, cells were seeded in 12-well plates at 100,000 cells per well. After overnight cultivation, cells were lysed in SDS sample buffer (30 mM Tris pH 6.8, 1 mM EDTA, 10% glycerol, 2% SDS, 0.1% bromophenol blue, 5% beta-mercaptoethanol) and heated to 95°C for 5 min. Denatured samples were subjected to SDS-PAGE and blotted onto nitrocellulose membranes (Protran; Amersham, Freiburg, Germany) in a wet-tank using a Mini-Protean electrophoresis system (Bio-Rad, Hercules, CA, USA). After transfer, filters were blocked for 1 h at room temperature in PBS-T/MP (PBS with 0.1% Tween-20 and 5% skim milk powder) followed by overnight incubation with primary antibody in 50 mL tubes at 4°C on a roller (CAT, Staufen, Germany). Filters were washed three times in PBS-T (PBS with 0.1% Tween-20) followed by incubation with secondary antibody in 50 mL tubes at room temperature for 1 h on a roller. After three washes in PBS-T, the filters were reacted with HRP Juice (PJK Biotech, Kleinblittersdorf, Germany) and signals detected in a ChemoCam Imager (Intas, Göttingen, Germany).

As primary antibodies we used the mouse anti-T antigen monoclonal PAb108 (ABIN967412; antibodies-online GmbH, Aachen, Germany) diluted 1:500 in PBS-T/MP and a rabbit anti-actin (A2066; Sigma-Aldrich, St. Louis, MO, USA) diluted 1:1,000 in PBS with 5% bovine serum albumin. For detection of GFAP, GABBR2, GAD2 (GAD65/67) or Olig1, we used recombinant monoclonal antibodies derived from mouse hybridoma [28]. The plasmids (kind gifts from James Trimmer, UC Davis) encoding anti-GFAP (#114536; Addgene, Watertown, MA, USA), anti-GABBR2 (#114544), anti-GAD65/67 (#177474) or anti-Olig1 (#114540) were transfected into 293T cells and antibody containing supernatants were harvested after 6 d, passed through a 0.45μm filter and used undiluted or 1:5 diluted for detection. As secondary antibodies we used horseradish conjugated anti-mouse, anti-rabbit or anti-goat antibodies (Dianova, Hamburg, Germany) diluted 1:10,000 in PBS-T/MP.

### RNAseq and Bioinformatics

For RNA-isolation, cells were seeded in 12-well plates in duplicate samples. After 24 h, total cellular RNA was extracted using the TRIZOL reagent (Ambion, Austin, TX, USA) following the manufacturer’s instructions. Extracted total RNA was subjected to 50 bp single-end bulk RNA sequencing (Illumina HiSeq 4000) at the Integrative Genomics Core Unit (NIG), Department of Human Genetics, University Medical Center Göttingen. Data were demultiplexed using bcl2fastq and further controlled for sequencing quality using FastQC (Galaxy Version 0.72). After verifying data quality, alignment was performed against reference genome Mmul_10 (https://www.ncbi.nlm.nih.gov/datasets/genome/GCF_003339765.1/) [29]. Raw count profiles were extracted from aligned bam files using featureCounts, and normalized TPM values were calculated.

### Data deconvolution using external single-cell RNAseq dataset

To prepare the deconvolution of the bulk RNA sequencing experiments, the reference scRNAseq dataset from GSE127774 was collected and reanalyzed. In brief, the aligned scRNAseq matrices from the macaque brain cortical region were acquired and reconstructed using the R package Seurat v4 [30]. The data object was then reanalyzed using identical parameters from the original publication [31]. Major cortical cell populations including excitatory neuron (Ex Neuron), inhibitory neuron (Inh Neuron), astrocyte, oligodendrocyte precursor cell (OPC), mature oligodendrocyte, and microglia were identified, consistent with what had been described in the reference publication [31].

Because the reference scRNAseq dataset used human gene annotations for integrative analysis, to use it as a reference, official gene symbols from raw count profiles from bulk RNA sequencing experiments were first translated to matching human gene annotations using the biomaRt package [32]. Afterwards, bulk profiles were deconvoluted with the reference scRNAseq data using R package MuSiC [33].

### Immunofluorescence staining

Cells were seeded in 24-well plates on 12 mm coverslips at 80.000 cells/well. After growth overnight, cells were washed with PBS and subsequently fixated with 4% paraformaldehyde (ROTI®Histofix, Roth, Karlsruhe, Germany) for 15 min at RT, followed by two washes with PBS. Cells were then permeabilized with PBS/0.2% Triton X-100 followed by washing in PBS/0.1% Tween-20. After blocking with PBS/10% FCS/1% BSA for 30 min at 37°C, cells were incubated with primary antibody (cell culture supernatant containing recombinant monoclonal antibody against GFAP) for 30 min at 37°C. After three washes with PBS/0.1% Tween-20 cells were incubated with secondary antibody Alexa Fluor488 conjugated donkey anti-mouse IgG (1:1000 in blocking buffer; Invitrogen Carlsbad, CA, USA) for 30 min at 37°C. After two washes in PBS/0.1% Tween-20, and one additional incubation for 5 min in PBS/0.1% Tween-20 containing 0.5μg/mL DAPI, cells were mounted in Mowiol/DABCO [34].

### Transduction with retroviral pseudoparticles

Retroviral pseudoparticles bearing glycoproteins of different viruses were produced in 293T cells essentially as previously described [24,25]. Briefly, cells were transfected in T25 flasks (about 10^6^ cells/flask) by calcium phosphate precipitation with 6 μg vector MLV-luc, 3 μg MLV-gag-pol and 3 μg expression plasmid for the respective viral glycoprotein. After three days, supernatants were harvested, filtered through 0.45 μm filters and stored at -80°C.

For infection, cells were seeded in 96-well plates at 10,000 cells per well in a volume of 50μL/well. On the next day, 50 μl pseudovirus containing supernatant was added in triplicate samples. After 4–6 hours, an additional 100 μl medium was added and cells were incubated for 3 days until harvest. For this, cells were lysed in 50 μl Luciferase Cell Culture Lysis Reagent (Promega, Madison, WI, USA) and luciferase activity was measured using Beetle-Juice (PJK Biotech, Kleinblittersdorf, Germany) as substrate and a Plate Chameleon V (Hidex, Turku, Finland) microplate reader.

### Induction of interferon system

To analyze the IFN response, cells were seeded in 12-well plates at 100,000 cells per well. On the next day, cells were either treated with IFN or infected with VSV ncp*, which has been reported to induce high levels of IFN [23]. For IFN treatment, universal type I IFN-α (pan-Interferon; 11200–2, PBL Assay Science, Piscataway), a chimeric interferon constructed from human IFN alpha A and alpha D [35] was added at 100 U/mL. Infections with VSV ncp* were carried out at MOI 0.1. After 24 h, cells were harvested for RNA isolation and quantitative PCR.

### Virus replication and titration

To assess virus replication, cell lines were seeded in 24-well plates at 50,000 cells/well and infected on the next day with either ZIKV or PaHV2 at an MOI of 1. After removing medium, 500 μl inoculum was added and cells were incubated at 37°C for 1 h. Then the inoculum was removed and cells were washed with phosphate-buffered saline (PBS). Finally, 500 μl fresh culture medium was added. Cell culture supernatant was harvested after 1 and 72 h, cleared from floating cells by centrifugation at 1,500 × g for 5 min and frozen at -80°C.

For titration of herpesviruses by plaque assay, we used a published protocol [36,37]. To measure titers of ZIKV we modified a focus formation assay originally developed for IAV [24,38]. In brief, we seeded Vero76 cells in 96-well plates at 20,000 cells/well. On the next day, tenfold dilutions of virus samples were prepared and cells were inoculated with 100 μL virus-containing culture supernatants. After incubation for 1 h at 37°C, the inoculum was replaced by 100 μL overlay medium (DMEM containing 0.5% methyl cellulose) and incubated for 3 d at 37°C. The overlay medium was then removed and cells were washed in PBS and fixated with ice-cold methanol for 10 min at -20°C. After removal of methanol, cells were dried and then rehydrated with PBS-T (PBS, 1% Tween-20) followed by quenching (PBS, 0.5% triton X-100, 20mM glycin) and blocking (PBS, 0.5% triton X-100, 1% bovine serum albumin (BSA)). For detection of ZIKV E protein expression, cells were incubated with 50 μl/well hybridoma 4G2 supernatant for 30 min at 37°C. Cells were then washed three times with PBS-T followed by 50μl secondary anti-mouse horseradish peroxidase (HRP)-conjugated antibody (1:1,000 in blocking buffer, Dianova, Hamburg, Germany) and again three washings with PBS-T. Cells were then reacted with TrueBlue peroxidase substrate (Seracare, Milford, MA, USA) until blue foci developed. Foci were counted and titers calculated and expressed as focus forming units per milliliter (ffu/mL).

### Quantitative real-time PCR

For RNA isolation, the RNeasy Mini Kit (Qiagen, Hilden, Germany) was used according to the protocol of the manufacturer and RNA was finally eluted in a volume of 25 μl RNase-free water. Subsequently, 1 μg RNA was treated with 0.5 U RNase-free DNase I for 10 min to remove contaminating DNA. The reaction was stopped by addition of EDTA (final concentration 5 mM) and heating to 75°C for 10 min. The SuperScript™ III First-Strand Synthesis System (Thermo, Waltham, MA, USA) was used for cDNA synthesis from 8 μl of DNase-digested RNA using random hexamers. Quantitative PCR was performed using 1 μl of the cDNA preparation and a QuantiTect SYBR Green PCR kit (Qiagen, Hilden, Germany) on the Rotorgene Q platform (Qiagen, Hilden, Germany). Primers against IFNB1 (forward 5’-CAGCAATTTTCAGTGTCAGAAGC-3’, reverse 5’- TCATCCTGTCCTTGAGGCAGT-3’), MX1 (forward 5’- TTCAGCACCTGATGGCCTATC-3’, reverse 5’- TGGATGATCAAAGGGATGT-GG-3’), and the housekeeping gene 18S rRNA (forward 5’-GATCCATTGGAGGGCAAGTCT-3’, reverse 5’-CCAAGATCCAACTACGAGCTT-3’) have been described previously [39–41]. The 2^-ΔΔCT^ method [42] was used to calculate induction of MX1 and IFNB1 expression by using 18S rRNA as reference gene.

### Microscopy

Brightfield images were taken on a Zeiss Axio Observer Z1/7 confocal microscope at 10x magnification (10x/0.45 Plan-Apochromat objective) using the electronically switchable illumination and detection module (ESID) and ZEN software. For figure preparation, images were subsequently cropped and additionally processed (adjustment of brightness, scale bar) using ImageJ/Fiji [27,43]. Immunofluorescence images were taken at 20x magnification (20x/0,75 Plan-Apochromat) using ZEN software. For figure preparation, images were equipped with a scale bar using ImageJ/Fiji.

## Results

### Establishment of immortalized rhesus macaque cell lines from neuronal tissues

**Fig 1:**
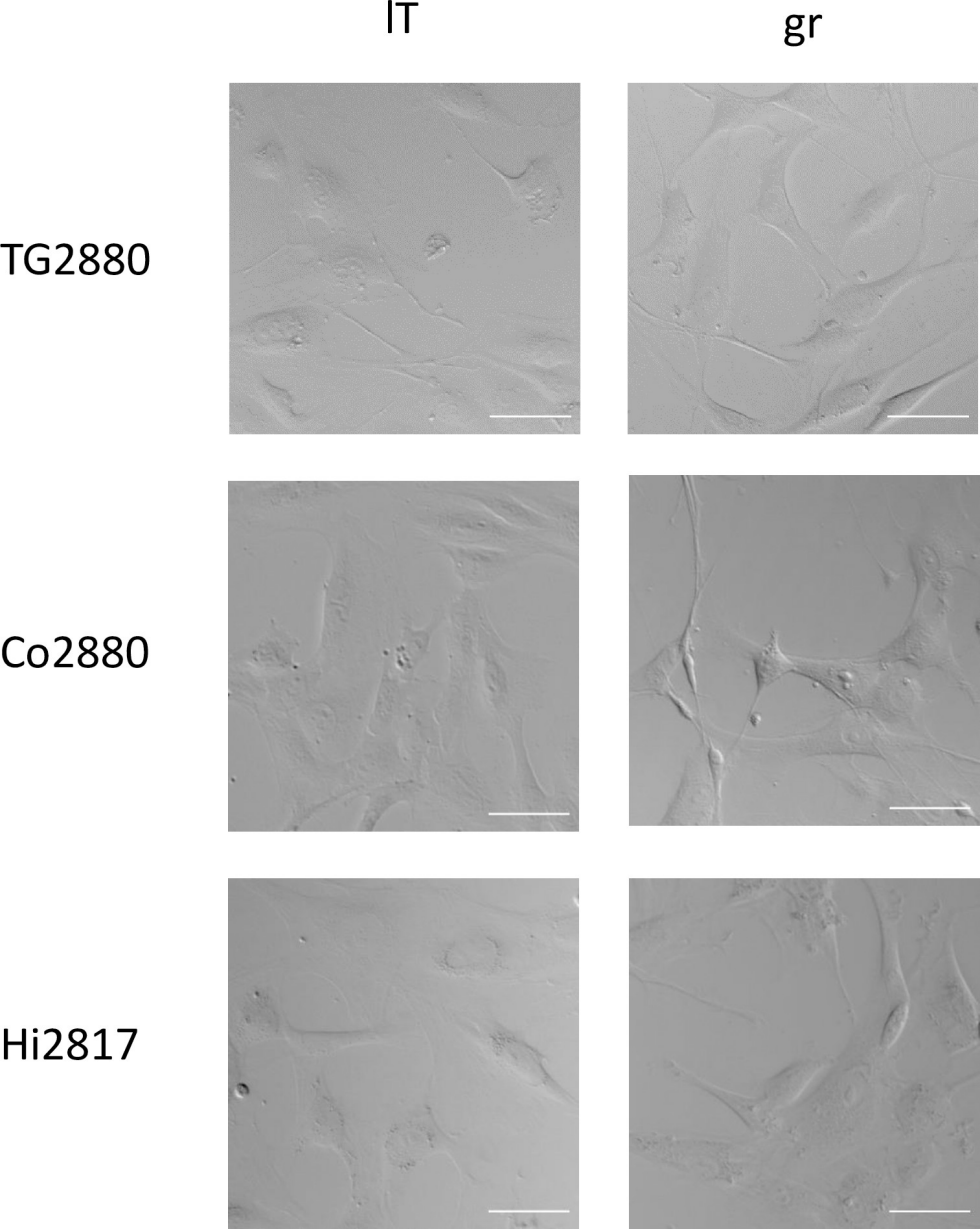
Morphology of rhesus macaque cell lines. Cells were seeded in 6-well plates and bright field images taken at 10x magnification. White scale bars indicate 50 μm.

**Fig 2:**
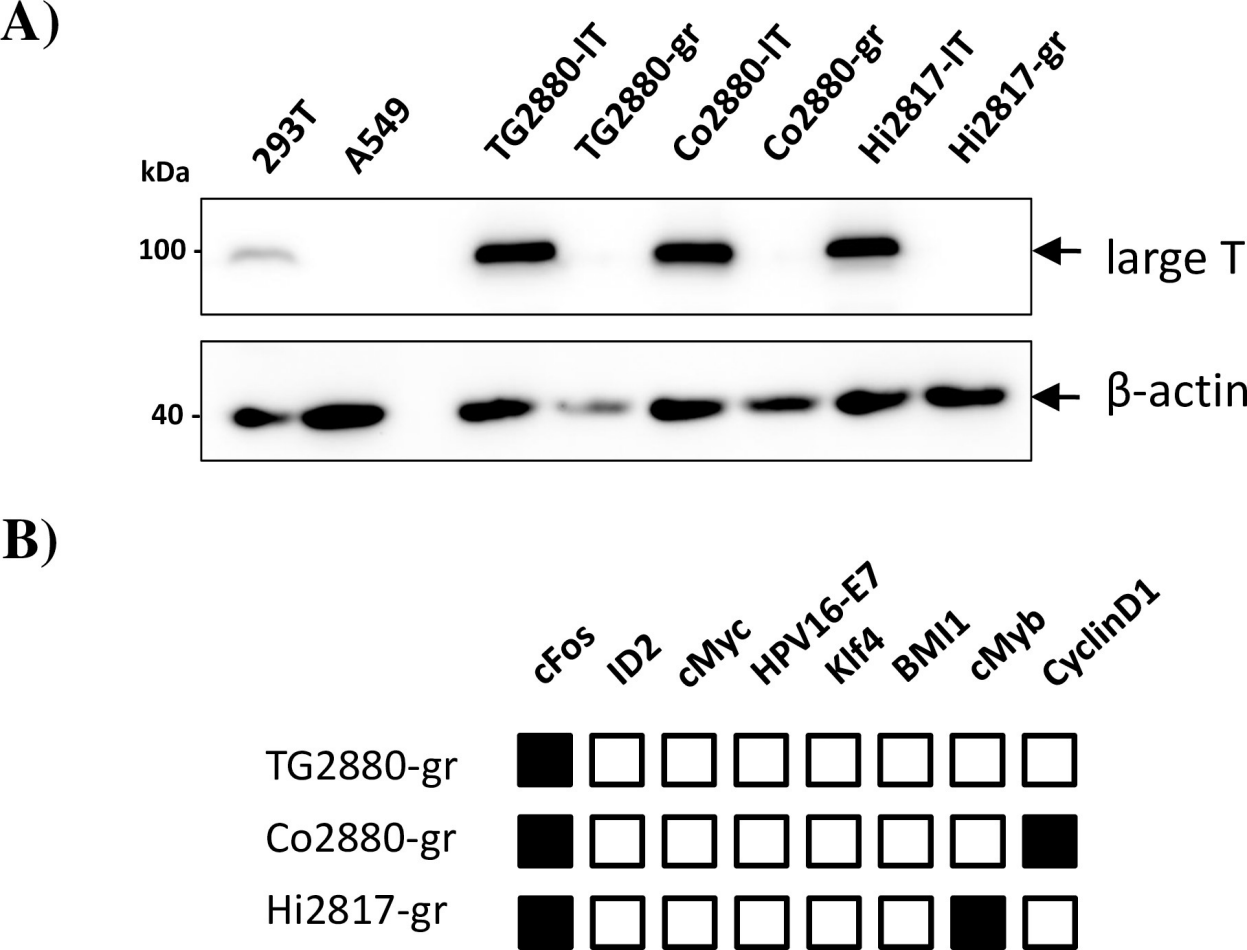
Characterization of marker expression in rhesus macaque cell lines. (A) Lysates of the indicated cell lines were analyzed by immunoblot for expression of the immortalization gene large T. Human cell lines 293T and A549 served as positive and negative controls, respectively, for large T expression. Detection of β-actin (ACTB) in the same lysates on a separate blot served as expression control. Similar results were obtained in a separate experiment. (B) Cell lines transduced with a mix of immortalization genes were analyzed by PCR for the presence of integrated transducing vector by a combination of vector specific and gene specific primers. Detection of a transduced gene is indicated by black boxes, while white boxes indicate absence.

To establish cell lines from neuronal tissues of rhesus macaques, we dissociated tissue samples from trigeminal ganglia, cortex and hippocampus of two adult male animals (6 and 7 years old, animals 2817 and 2880, respectively) and established primary cultures. The cell lines were termed *Macaca mulatta* (Mamu) trigeminus ganglion (TG), Cortex (Co) and Hippocampus (Hi), respectively. After few passages, cells were transduced with lentiviruses expressing SV40 large T (lT), or a mixture of genes shown to immortalize cells [27]. To avoid TRIM5α-mediated restriction of HIV-based vectors we used a modified lentiviral system, which included a chimeric SIV/HIV-Gag protein [18]. By selection with puromycin or hygromycin, resistant cell cultures were established after transduction with either large T (“lT”) or a mixture of immortalizing genes (termed “gr”) and termed MamuTG2880-lT, MamuTG2880-gr, MamuCo2880-lT, MamuCo2880-gr, MamuHi2817-lT and MamuHi2817-gr. Throughout this work we skip the prefix “Mamu” since it is clear that these cell lines are derived from rhesus macaques (*Macaca mulatta*). Cells had a short spindle-like shape as seen in mesenchymal cells and a number of cells showed protrusions (Fig 1). Cells were successfully passaged for multiple passages. However, after prolonged passaging (up to 50 population doublings) we observed that “gr” cell lines ceased to grow, rendering them not fully immortalized. In contrast, the “lT” cell lines have so far grown to 70–90 population doublings. Expression of large T was confirmed by immunoblot (Fig 2A), using 293T cells as positive and A549 cells as negative control, while the transduction with genes from the immortalization mixture was analyzed by PCR. Only a subset of the transduced genes could be detected in the established cell lines. All transduced cell lines contained the c-Fos gene, while Co2880-gr and Hi2817-gr additionally contained the Cyclin D1 and c-Myb genes, respectively (Fig 2B).

### RNAseq analysis reveals astrocyte lineage of MamuCo2880-gr cells

**Fig 3:**
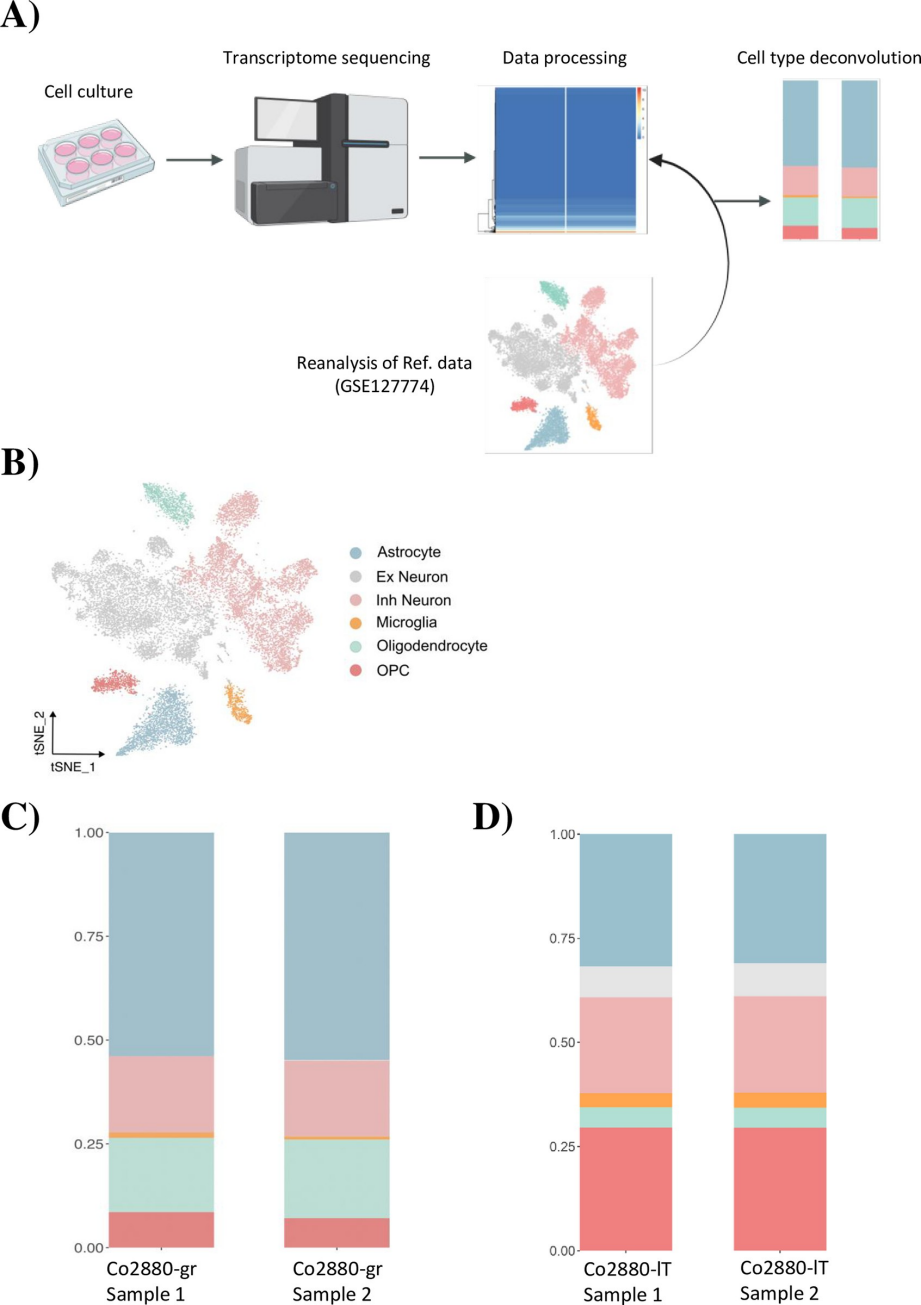
Transcriptome sequencing and cell type deconvolution. (A) Workflow used for transcriptome sequencing and deconvolution analysis. Bulk sequencing profiles were processed to a gene expression matrix, and relevant cell types were deconvoluted by reference to a scRNAseq dataset from rhesus macaque brain tissue (GSE127774). (Created with BioRender.com). (B) Reanalysis of reference scRNAseq data recognized major CNS cell populations and was used for downstream deconvolution analysis. (C) and (D) Stack barplots show putative proportions of different cell types in deconvoluted bulk RNA sequencing experiments for Co2880-gr (C) and Co2880-lT (D) cells.

To gain more insight into the origin of the cell lines, we chose the cell line Co2880-gr, which seemed to show more protrusions than the other cell lines, indicating a more differentiated state. In addition, we chose to analyze Co2880-lT, which showed a more spindle-like morphology. Total RNA isolated from these cell lines was subjected to bulk RNAseq analysis (Fig 3A). Referring to publicly available single-cell resolution RNAseq data from rhesus macaques cortical region (GSE127774) [31] (Fig 3B), we used a deconvolution approach to identify which cell type is best represented by our cell lines [33]. Deconvolution demonstrated that in both independent RNA samples of Co2880-gr cells, cells of the astrocyte lineage represented the most abundant cell population (Fig 3C). In contrast, for the Co2880-lT cell line the deconvolution analysis revealed equal populations of cells of astrocyte, inhibitory neuron or oligodendrocyte origin (Fig 3D). Thus, we obtained large T or FOS-expressing cell lines from all three tissues and found that the FOS expressing cell line Co2880-gr largely represented the astrocyte lineage.

### Characterization of marker expression in cell lines from neuronal tissue

**Fig 4:**
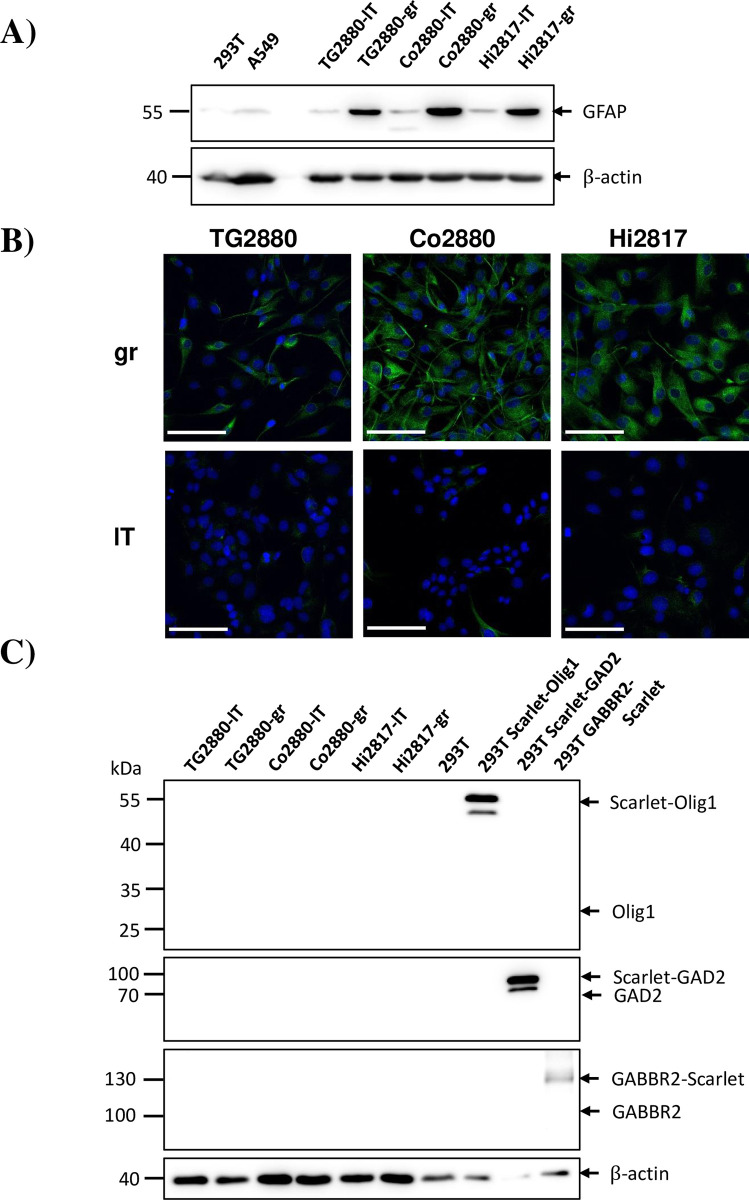
Expression of the astrocyte marker GFAP in rhesus macaque cell lines. (A) Lysates of immortalized cell lines were analyzed by immunoblot for expression of glial fibrillary acidic protein (GFAP). Detection of β-actin (ACTB) in the same lysates on a separate blot served as expression control. Similar results were obtained in a separate experiment. (B) Immunofluorescence staining of immortalized cell lines with an antibody against GFAP (green). Nuclei were stained with DAPI (blue). Scale bar indicates 100 μm. Similar results were obtained in a separate experiment. (C) Lysates of immortalized cell lines were analyzed by immunoblot for expression of oligodendrocyte (Olig1) or inhibitory neuron (GAD2, GABBR2) markers. As control, rhesus genes fused to scarlet fluorescent protein were expressed in transfected 293T cells. Expected molecular weights for the respective proteins are indicated by arrows. Detection of β-actin (ACTB) in the same lysates on a separate blot served as expression control. Similar results were obtained in a separate experiment.

To address cell line identity more directly, we first used detection of marker genes by immunoblot and immunofluorescence microscopy. Using an antibody directed against glial fibrillary acidic protein (GFAP) we were able to demonstrate strong expression in all “gr” cell lines, while expression in “lT” cell lines was much lower (Fig 4A). Immunofluorescence staining and microscopy of “gr” cell lines showed an uniform expression of GFAP in almost all cells, while in the “lT” cell lines only individual cells showed clear GFAP staining (Fig 4B). To address the possibility that additional cell types, notably inhibitory neurons and oligodendrocytes, were present in the cultures, we performed immunoblot analysis using antibodies directed against GAD2 or GABBR2 (markers for inhibitory neurons) or OLIG1 (marker for oligodendrocytes). No signals could be detected for any of these markers in our cell lines (Fig 4C). As all antibodies were directed against human proteins, we expressed the corresponding rhesus macaque proteins fused to Scarlet fluorescent protein and demonstrated robust antibody reactivity (Fig 4C). These results corroborate the RNAseq data discussed above and suggest that the “gr” cell lines generated are likely of astrocyte origin.

### Evidence that immortalized cell lines from neuronal tissue have a functional IFN system

**Fig 5:**
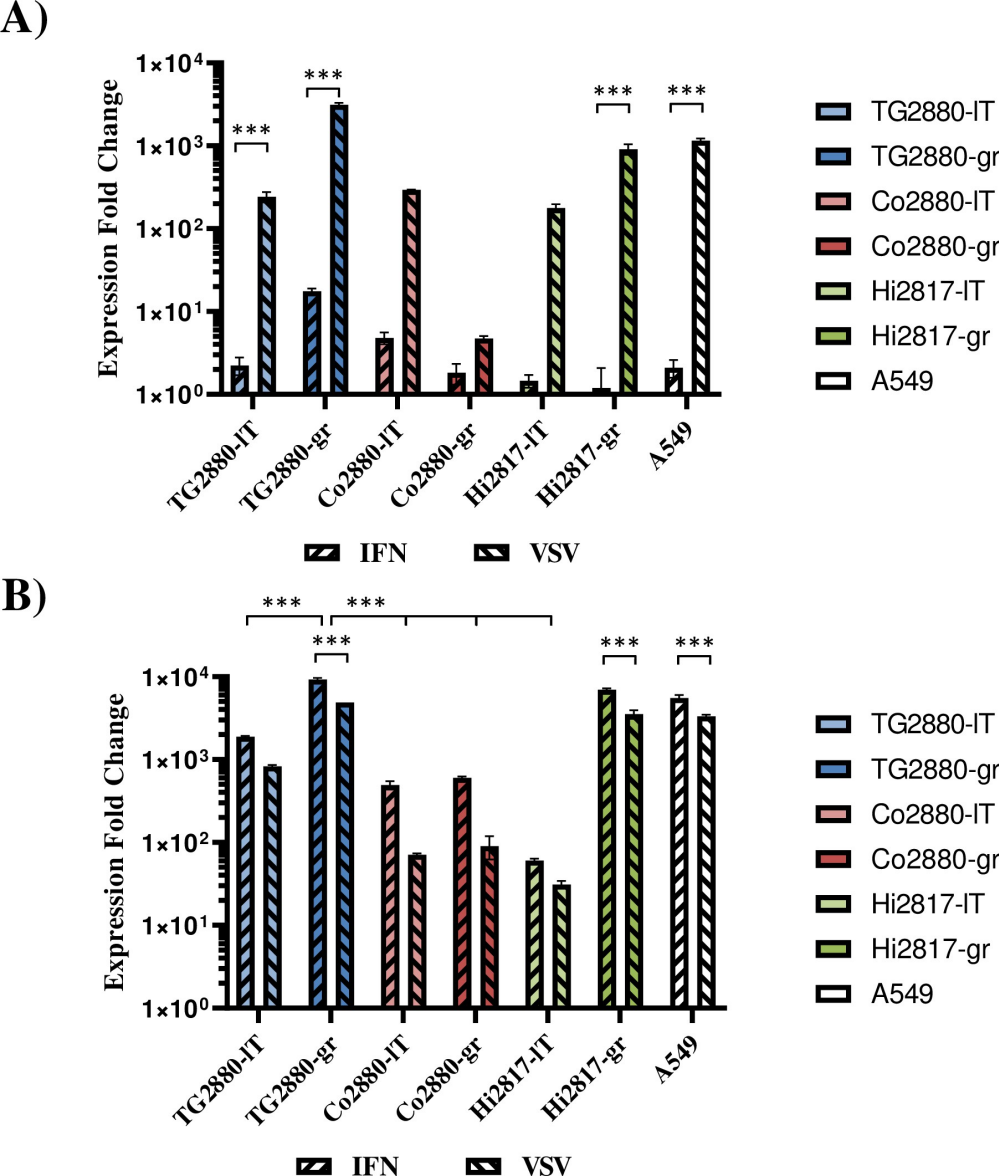
Evidence that the rhesus macaque cell lines have a functional interferon system. Cells seeded in 12-well plates were either treated with 100 U/mL pan-IFN or infected with VSV ncp* (MOI 0.1). Untreated cells served as control. Cells were harvested after 24 h for RNA isolation. Expression of (A) interferon beta (IFNB1) or (B) MX1 was analyzed by quantitative RT-PCR. Transcript levels were normalized against 18S rRNA transcript levels and expression fold change was calculated with respect to control cells. The results of a representative experiment carried out with technical triplicates are shown. Error bars indicate standard deviation. Similar results were obtained in a separate experiment. Statistical significance was tested by two-way ANOVA: *, p≤0.05; **, p≤0.01; ***, p≤0.001.

Next, we characterized whether the cells expressed interferon β1 (IFNB1) or the interferon stimulated gene (ISG) MX1 in response to treatment with IFN or virus infection. For this, cells were either treated for 24 h with 100 U/mL pan-IFN, a chimeric human IFN alpha, or infected with VSV ncp*, which strongly induces IFN [23]. Quantitative RT-PCR was used to analyze induction of IFNB1 or MX1. As positive control, we included the human lung cell line A549, which has an intact IFN system [44]. Strong induction of IFNB1, comparable to the A549 cell line, was observed in most cell lines after infection with VSV (Fig 5A), while treatment with pan-IFN had only minor effects. However, pan-IFN strongly induced the ISG MX1 (Fig 5B) in all cell lines, although the actual levels of induction differed between cell lines. Infection with VSV also induced MX1 to levels similar to those measured upon pan-IFN treatment. These findings suggest that major pathways of the IFN system are intact in all newly established cell lines.

### Immortalized cell lines from neuronal tissue are susceptible and permissive to virus infection

**Fig 6:**
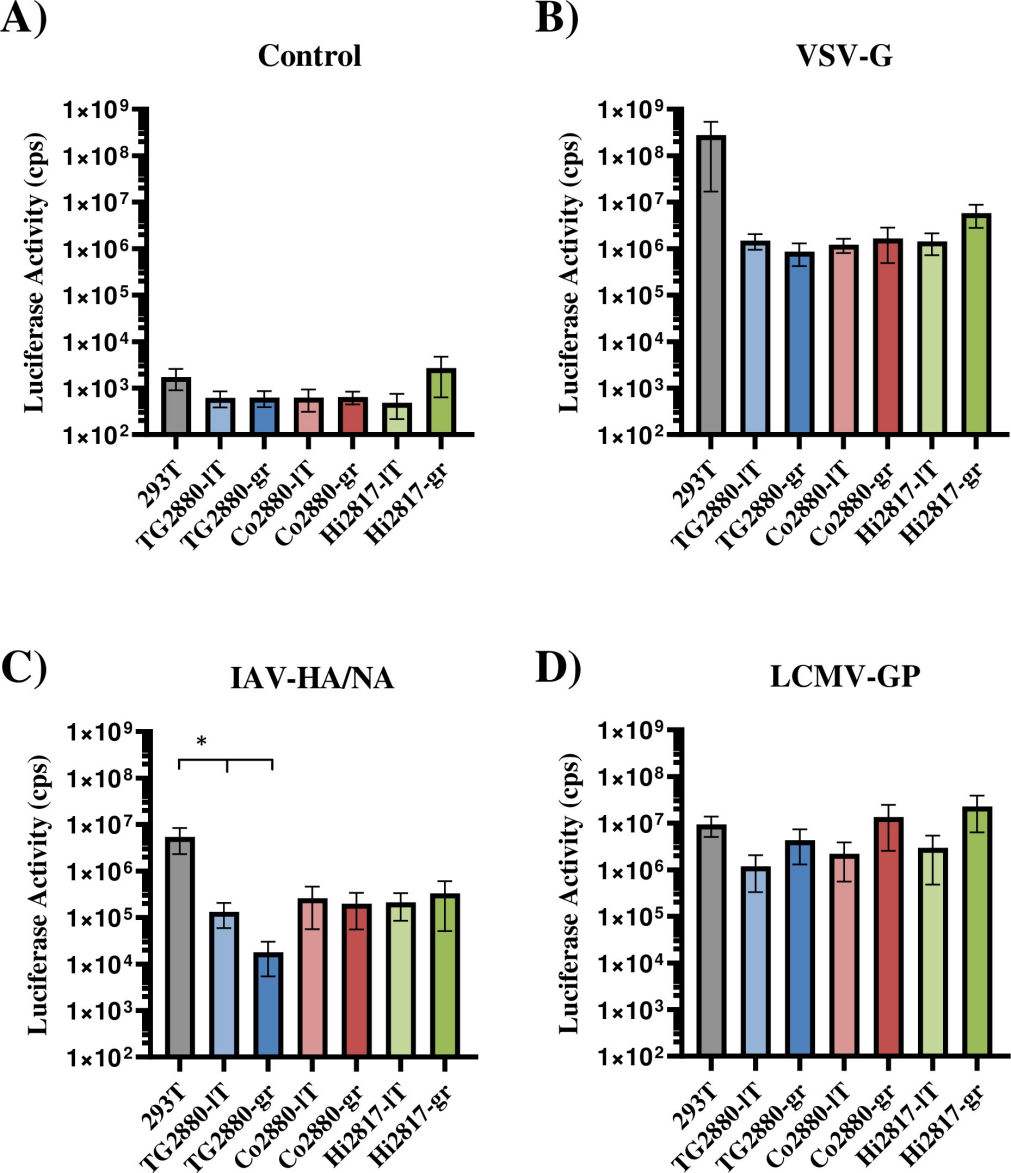
The rhesus macaque cell lines are susceptible to entry driven by several viral glycoproteins. The cell lines were seeded in 96-well plates and transduced in triplicates with MLV pseudoparticles encoding firefly luciferase and bearing the viral glycoproteins VSV-G (B), IAV-HA/NA (C) and LCMV-GP (D). Pseudoparticles without viral glycoprotein (Control) served as negative control (A), while transduction of 293T cells was used as positive control. Cell lysates were harvested after 72 h and luciferase activities determined. The average of four independent experiments performed with technical triplicates is shown. Error bars indicate standard error of the mean. Statistical significance was tested by two-way ANOVA: *, p≤0.05; **, p≤0.01; ***, p≤0.001.

We then proceeded to evaluate the susceptibility and permissiveness of the cells to infection by several viruses. To test susceptibility, we used retroviral pseudotypes bearing the glycoproteins of Indiana vesiculovirus (VSV), influenza A virus (IAV) or Lymphocytic choriomeningitis virus (LCMV). As a negative control, we used viral particles bearing no glycoprotein, while 293T cells served as positive control since this cell line is known to allow entry driven by all viral glycoproteins tested. The rhesus macaque cell lines allowed for entry driven by all glycoproteins analyzed although with different efficiency, while entry of particles bearing no glycoprotein was in the background range of the assay (Fig 6). Thus the LCMV glycoprotein facilitated entry into the rhesus macaque cell lines and 293T cells with similar efficiency, while macaque cell entry driven by the VSV and IAV glycoproteins was reduced by more than 10-fold as compared to 293T cell entry.

**Fig 7:**
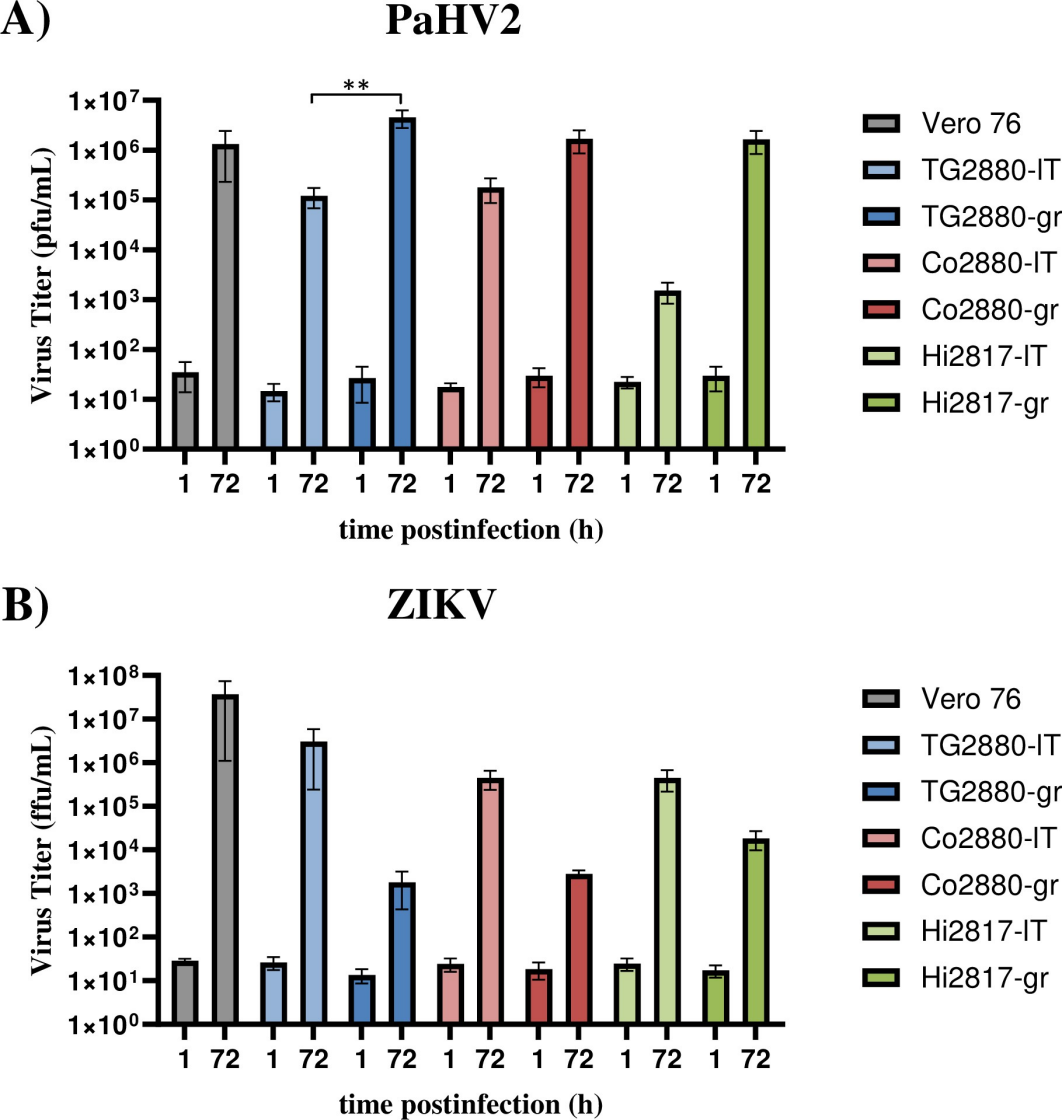
The rhesus macaque cell lines support growth of Papiine alphaherpesvirus 2 and Zika virus. For infection with (A) Papiine alphaherpesvirus 2 (PaHV2) or (B) Zika virus (ZIKV) strain MR766, cells were seeded in 24-well plates. Vero76 cells served as positive control. Cells were infected in triplicates at an MOI of 1. Supernatants were harvested at 1 h, as baseline, and at 72 h postinfection. Infectious virus titers were determined by plaque assay (PaHV2) or focus formation assay (ZIKV) on Vero76 cells and virus titers expressed as plaque forming units (pfu) or focus forming units (ffu), respectively. The average of three independent experiments performed with technical triplicates is shown. Error bars indicate standard error of the mean. Statistical significance tested by two-way ANOVA: *, p≤0.05; **, p≤0.01; ***, p≤0.001.

Finally, we tested the permissiveness of the rhesus macaque cell lines to infection with a DNA and a RNA virus. As DNA virus we employed the primate simplexvirus Papiine alphaherpesvirus 2 (PaHV2), which naturally infects NHP, and as RNA virus we used ZIKV, a human pathogen for which rhesus macaques serve as animal model [45,46]. We determined titers at 72 hours post infection, since both viruses achieved the plateau at this time point in single-step growth curves for several cell lines, including Vero76 cells, which also served as positive control. Titers right after infection (1 h) served as a background control. For PaHV2 titers comparable to Vero76 cells were achieved, especially for “gr” cell lines, while the large T-immortalized cell line from hippocampus (Hi2817-lT) yielded reduced titers (Fig 7A). Titers obtained after infection with ZIKV were generally reduced compared to Vero76 cells and this was most pronounced for the cell lines established with the immortalization mix (“gr”) (Fig 7B).

## Discussion

We report the generation and analysis of a total of six cell lines originating from cortex, hippocampus or trigeminal ganglia of two male rhesus macaques. Major pathways of the IFN system were intact in these cell lines, they allowed entry driven by the glycoproteins of diverse viruses and were permissive to infection by PaHV2 and ZIKV.

The cell lines were generated by either transduction of SV40 large T (“lT” cell lines), a well known immortalization gene, or by employing mixtures of transgenes (“gr” cell lines). Expression of large T was confirmed by immunoblot. For cell lines transduced with a mix of transgenes known to immortalize cells, genomic PCR demonstrated the uniform presence of c-Fos in these cell lines, while Co2880-gr also carried Cyclin D1 and Hi2817-gr harbored c-Myb. The fact that only few transgenes were detected in the established lines has been previously reported and was therefore not unexpected [27]. Since expression of the transgenes was linked to that of hygromycin, which was used for successful cell selection, we assume that the transgenes are actively expressed. In fact, RNAseq analysis of the Co2880gr cells revealed high levels of FOS and CCND1 expression.

The cell lines demonstrated a spindle-like mesenchymal morphology with protrusions, which were more prominent in the “gr” lines. RNAseq of cell line Co2880-gr followed by deconvolution analysis based on scRNAseq data from rhesus macaque brains demonstrated high similarity to cells of the astrocyte lineage. In keeping with this finding, Co2880-gr cells expressed the astrocyte marker GFAP, as determined by immunoblot and immunofluorescence microscopy. Similarly, GFAP was detected in all cell lines expressing FOS (“gr” cell lines) but not large T, suggesting that expression of FOS might better preserve the astrocyte phenotype. Our cell lines did not perfectly reflect astrocyte cells in the deconvolution analysis, which may be explained by alterations imposed on the cells by prolonged cell culture and transgene expression. In addition, the cells have not yet been cloned and it cannot be excluded that they still contain cells of other lineages, which would result in gene expression patterns overlapping with other lineages. However, by immunoblot we were unable to detect expression of marker genes for other lineages as suspected from the deconvolution analysis. The identity of the large T transduced cell lines is currently less clear and needs further investigation. Immunofluorescence microscopy revealed that a fraction of cells also expressed GFAP, whereas no expression of marker genes of other lineages was detected by immunoblot. While large T has previously been used for immortalization of astrocytes [47–49], transduction of the initial mixed cell population with large T may have led to selection of different non-astrocyte cell lineages.

The IFN system poses an important innate defense against virus infection, which can efficiently limit viral replication. As many cell lines have been derived from cancer cells, they frequently harbor defects in the IFN system. For instance, Vero cells are often used for efficient virus amplification and their locus for IFN alpha and beta has been deleted [50]. For the cell lines analyzed here, we could demonstrate that major aspects of the IFN system, including sensing of virus and induction of ISG expression, are intact, as evidenced by strong IFN beta induction upon VSV infection and strong induction of the ISG MX1 upon IFN treatment or viral infection.

Our brain-derived rhesus macaque cells lines allowed entry driven by the glycoproteins from representatives of the Rhabdoviridae (VSV), Orthomyxoviridae (IAV) and Arenaviridae (LCMV), all of which have been reported to cause infections of the brain [51–55], while also experimental infection of astrocytes has been demonstrated for VSV [56,57], IAV [58–60] and LCMV [61]. However, entry driven by the IAV glycoprotein was less efficient than for human 293T cells. In addition, we demonstrated productive infection with the primate herpesvirus PaHV2, as well as ZIKV (Flaviviridae). Since astrocytes are target cells for VSV [56,57], IAV [58–60,62], ZIKV [63,64] and Simplexviruses [65,66], our astrocyte cell lines may be interesting tools for in vitro analysis of neurotropic viruses.

## Conclusions

In summary, we established six rhesus macaque brain-derived cell lines with an intact IFN system. Three of the cell lines originated from the astrocyte lineage. The cell lines support infection with a primate herpesvirus and ZIKV and likely other viruses and may therefore be valuable tools in translational research or comparative infection research.

## Supporting information

S1 Fig Original blot images. (PDF)

S1 Table Minimal data sets. (XLSX)
