# Supplementary material for: Development of rhesus macaque astrocyte cell lines supporting infection with a panel of viruses
Source: PLoS One. 2024 May 14;19(5):e0303059. doi: 10.1371/journal.pone.0303059 (PMC11093292; doi:10.1371/journal.pone.0303059)

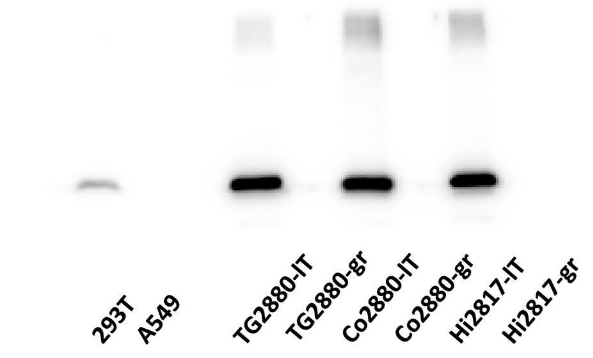

Anti large T

Anti β-actin

Fig. 2

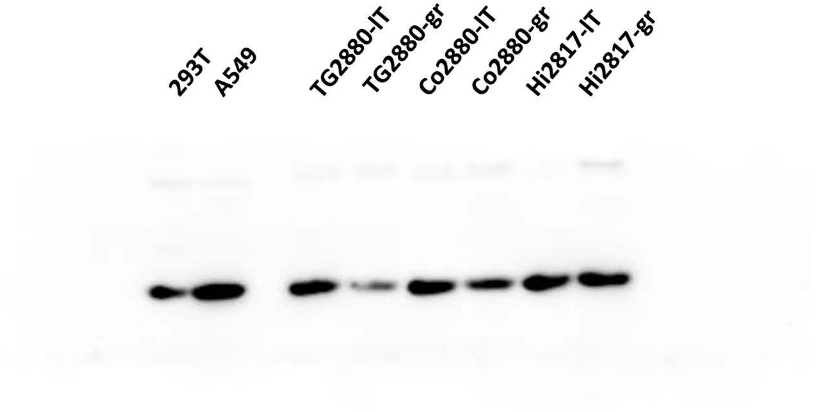

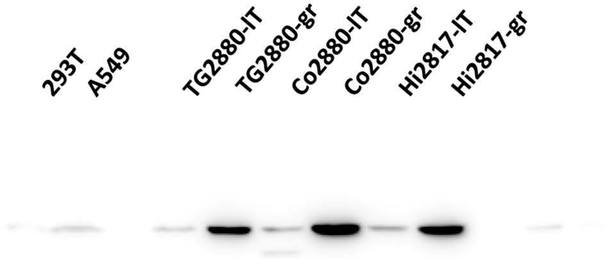

Anti-GFAP
Anti-β-actin

Fig. 4A

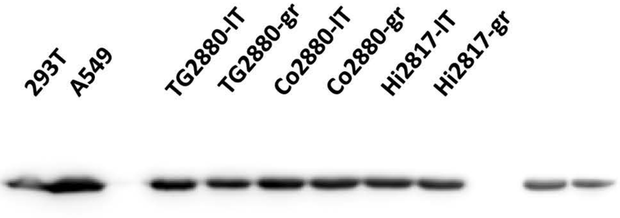

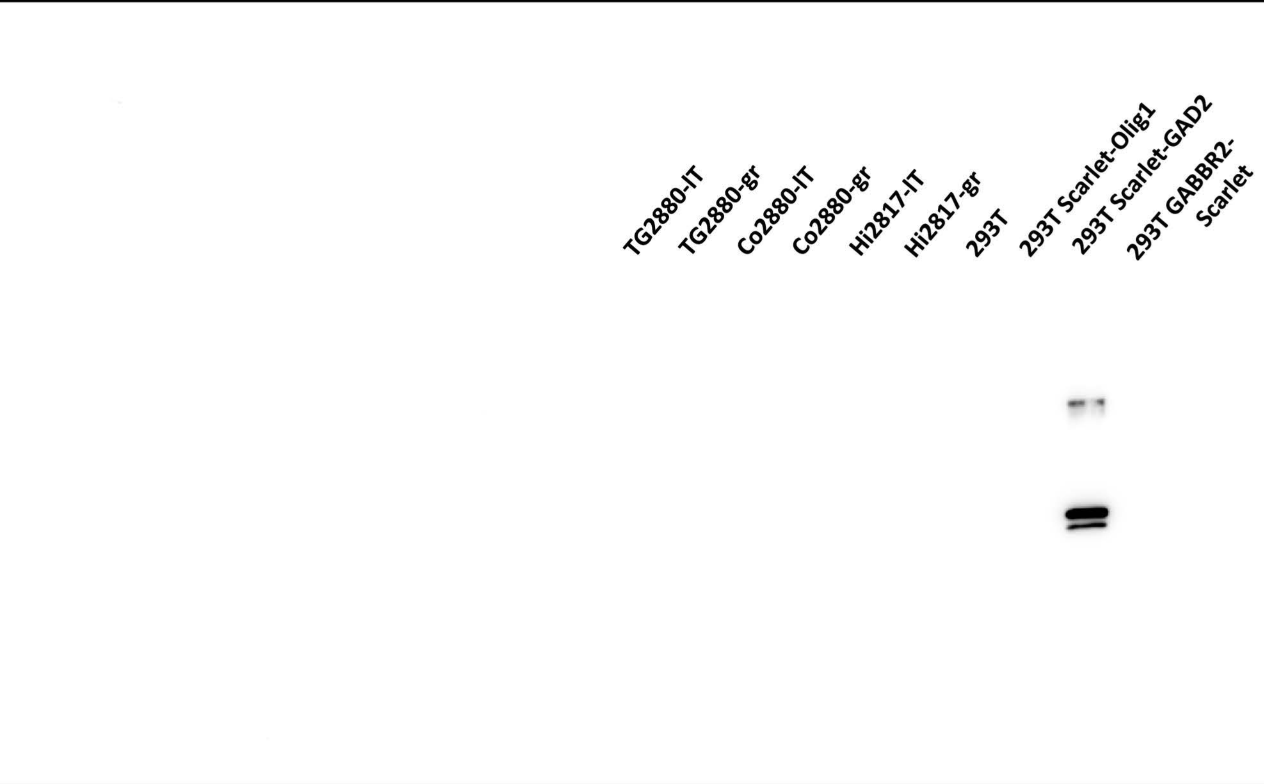

Anti-GAD2

Fig. 4C

Anti-Olig1

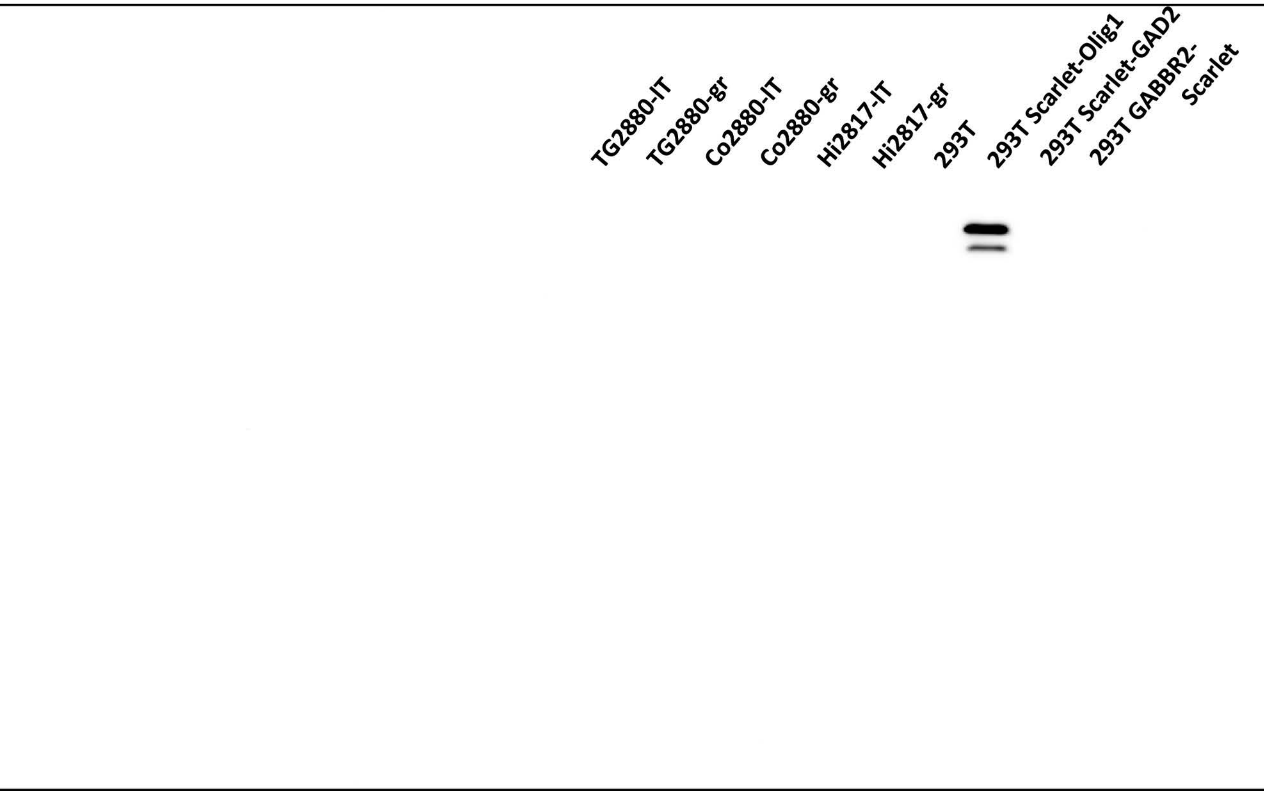

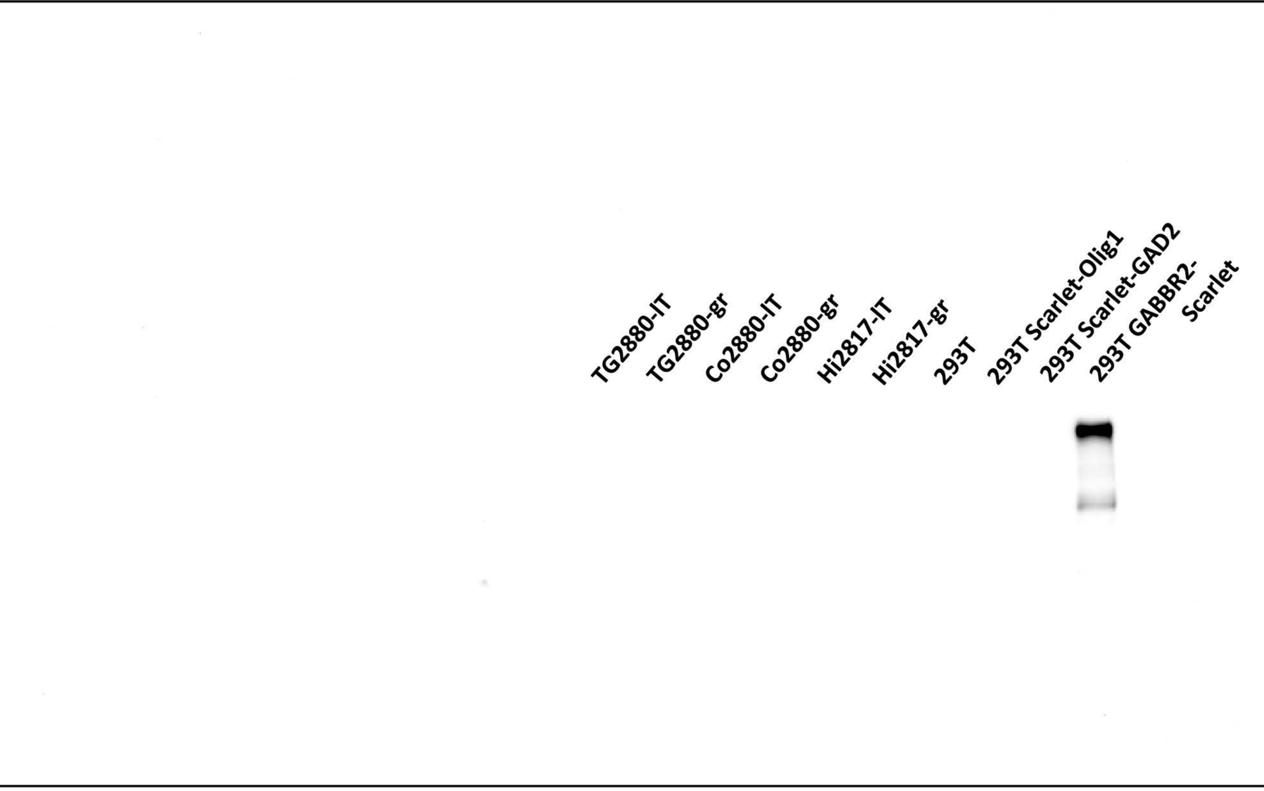

Anti-GABBR2

Fig. 4C

Anti-β-actin

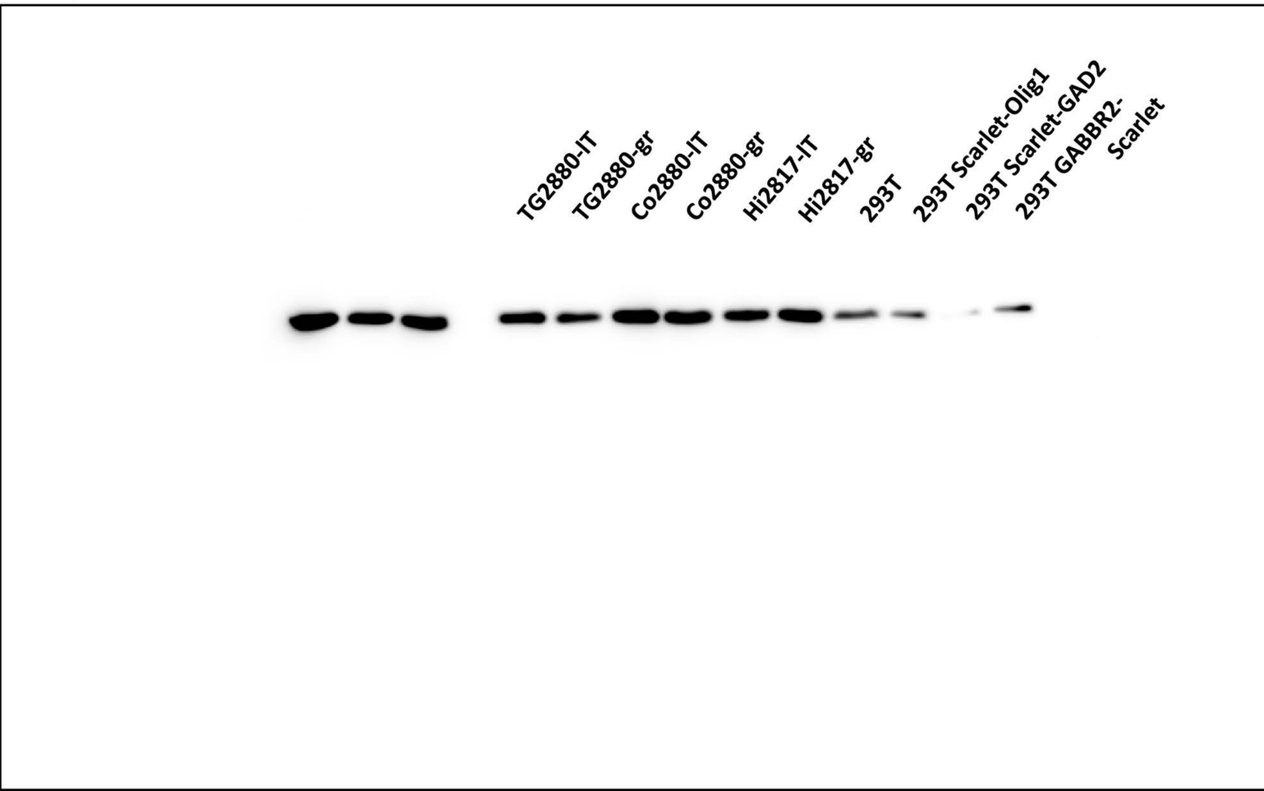

Supplement: S1 Fig — (PDF) [file pone.0303059.s001.pdf]
